# Risk factors related to liver injury in non-Intensive Care Unit admitted patients infected with COVID-19: A retrospective study of 102 patients

**DOI:** 10.22088/cjim.11.0.520

**Published:** 2020

**Authors:** Amir Sadeghi, Pegah Eslami, Arash Dooghaie Moghadam, Ali Pirsalehi, Sajad Shojaee, Laya Jalilian Khave, Ghazal Sanadgol, Taha Hasanzade, Dorsa Shirini, Hamid Asadzadeh Aghdaei, Saeed Abdi, Mohammad Reza Zali

**Affiliations:** 1 Gastroenterology and Liver Diseases Research Center, Research Institute for Gastroenterology and Liver Diseases, Shahid Beheshti University of Medical Sciences, Tehran, Iran; 2 Basic and Molecular Epidemiology of Gastrointestinal Disorders Research Center, Research Institute for Gastroenterology and Liver Diseases, Shahid Beheshti University of Medical Sciences, Tehran, Iran

**Keywords:** COVID-19, liver injury, liver function tests, prognostic factor.

## Abstract

**Background:**

COVID-19 targets the liver and there is no available data about liver injury due to mild to moderate form of COVID-19. In this study, we evaluated the risk factors associated with liver injury in NON-ICU admitted COVID-19 patients.

**Methods:**

in this retrospective study, 102 eligible adult participants admitted in the ward were included. The patients with previous history of liver disease were excluded. The patients with AST or ALT or bilirubin more than normal ranges were allocated in liver injury group and patients with normal ranges of them were categorized in non-liver injury. Characteristics and laboratory data were analyzed between these two groups.

**Results:**

The mean age of the population was 55.13± 17.02 years old. The most common symptom was fever (45.8%). The most frequent co-morbidity was hypertension (25%). 65 patients had liver injury (63.72%). CRP were significantly higher in liver injury group (P=0.01). Univariate analysis reported ALKP, and CRP was associated significantly with liver injury (P=0.04, OR= 1.003, Cl 95%= 1.000-1.007; P=0.03, OR= 1.009, Cl 95%= 1.000- 1.017, respectively). No independent factor was detected in multivariate analysis. Based on the Spearman’s rank correlation coefficients CRP correlated significantly with AST (r=0.22, P=0.00). Moreover, neutrophil and CRP, correlated with ALT (r=0.01, P=0.90; r=0.23, P=0.02, respectively).

**Conclusion:**

No independent factor was detected to predict liver injury chance due to COVID-19. However, CRP had a significant association with it. It appears that the role of inflammatory pathways in liver damage was due to COVID-19.

Coronavirus disease 2019 (Covid-19) pandemic started at the end of December 2019 in Wuhan City, Hubei province, China (1). As of August, 13^th^, 2020, about 20,400,000 people struggled with this new type of coronavirus family all around the world, and also around 740,000 deaths have been recorded according to the 206 st situation report published by the World Health Organization (WHO)(2). Common signs and symptoms of COVID-19 are extremely similar to other viral pneumonia including fever, fatigue, headache, and dry cough, which occasionally evolve to acute respiratory failure (3-5). Also, COVID-19 can involve the liver organ and create liver injury conditions (6). COVID-19-related liver injury is characterized as a condition in which liver damage occurs among treatment or progression of disease in all COVID-19 patient, perhaps due to following suggested possible mechanisms: 1) immune-associated damage according to inflammatory condition 2) hepatic cells damage due to viral replication, 3) hypoxic hepatitis, 4) reactivation of patient s pre-exiting chronic liver diseases, and 5) drug-medicated liver damage (7). 

However, the whole mechanism still is in the state of hypothesis and more investigation is necessary to find accurate mechanism of liver damage following COVID-19 (7). In this regard, many studies have declared that in intensive care unit (ICU) patients, hepatic biochemistries in the serum of COVID-19 patients increased and liver damage occurred (8, 9). Moreover, a few studies were focused on ward-admitted patients showing the occurrence of liver damage in COVID-19 patients, although less analysis on mechanisms of involvement and contributed factors (10). Overall, further investigation is needed to declare how COVID-19 affects liver organ and builds up the liver injury. In the present retrospective study, we attempted to evaluate factors leading to liver injury among non-ICU patients to provide a better vision of hepatic damage mechanism in COVID-19 patients.

## Methods

**Patients: **On behalf of the Ministry of Health of Iran, Taleghani Hospital (affiliated to Shahid Beheshti Medical University) was selected as a referral center in Tehran, capital of Iran, to admit COVID-19 patients. This study was designed to evaluate the relationship between liver injury and other factors in COVID-19. In this retrospective study, the medical electronic records of 240 proving that COVID-19 patients between 22 February and 22 April in Taleghani Hospital were evaluated by two trained researchers. Patients were excluded from our study if they were admitted to ICU during their hospitalization or received any medications due to Covid-19 before hospital admission or represented the positive history of chronic liver disorders including viral infections, alcoholic hepatitis and liver malignancies, or their age was below 18 years. Finally, 102 patients were included in this study. The clinical records were gathered including age, sex, comorbidities, past medical histories, first admission sign, symptoms, and also the duration of hospitalization and laboratory data. Moreover, according to importance of eliminating fatty liver disease affect our study, patients’ co-morbidities which were related to fatty liver disease and metabolic syndrome such as body mass index (BMI), ischemic heart disease (IHD), hypertension (HTN) and diabetes, were specifically evaluated for all of our patients and also Patient ‘s Habitual history including smoking and alcohol consumption were gathered. Afterwards, the patients were divided into two groups, with and without the liver injury category according to the level of Aspartate transaminase (AST), Alanine transaminase (ALT) or Bilirubin during admission, and were compared with regard clinical presentation and characteristic. We considered those parameters due to analyzing hepatocellular damage patterns in Covid-19 patients. Moreover, liver enzyme levels were collected again at the time of discharge and covid-19 treatment. The present research was approved by the local Ethics Committee of Shahid Beheshti University of Medical Sciences (Code:IR.SBMU.RIGLD.REC.1399.029). The study adhered to the Declaration of Helsinki and other applicable laws, regulations, and guidelines.

**Definitions:**In our study, we defined a COVID19 proven patient as the one with a positive report of severe acute respiratory syndrome coronavirus 2 nucleic acid Real-Time Polymerase Chain Reaction (SARS-CoV-2 nucleic acid RT-PCR). For this purpose, the specimens derived from oropharyngeal swabs or sputum were used. Moreover, all patients with no positive history of liver injury who have had an abnormal level of aspartate aminotransferase (AST), alanine transaminase (ALT), or bilirubin due to infection of COVID-19 were included in the liver injury group.

**Statistical Analysis: **Descriptive information including categorical variables as percentages and continuous variables were reported using mean±SD. Continuous variables were compared using independent t-test when data were normally distributed. Otherwise, Mann-Whitney test has been used. Some quantitative factors, such as AST, ALT and Alkaline Phosphatase Test (ALK-P), were examined at the time of admission and after covid-19 treatment. Of course before that, a number of fatty liver and metabolic syndrome risk factors were investigated in both groups. Categorical variables were compared using the Chi-square test. Also Fisher’s exact test was used if the data were limited and the number of expected values was insufficient ​​in the cells. Spearman’s rank correlation coefficients between liver enzyme levels (AST and ALT) and clinical characteristics were used. In addition, logistic regression models were developed to identify independent risk factors of liver damage. SPSS 23.0 software was applied for all statistical analysis. Statistical significance was defined as P<0.05.

## Results

The hospitalization data were collected in Taleghani Hospital between 22nd February and 22nd April. After exclusion, 102 participants were allocated in our study. Of these participants, 40 were females and 62 were males. The mean age of patients in our study was 55.13±17.02 years old. Common symptoms among these patients were fever (45.8%), dyspnea (44%), and cough (40.5%). Diarrhea was the most common gastrointestinal feature among non-ICU patients (18.5%). The most frequent co-morbidity among our population included hypertension (25%), Coronary Heart Diseases (CHD) (19%), and Diabetes Mellitus (DM) (15.5%). Among the non-ICU cases of our study, including 102 patients, 65 (63.72%) participants were detected with liver injury. AST increased to more than 40 mg/dl in 47.1% of the study population. ALT increased up to 40 mg/dl 43.1% and total bilirubin only in 18.6% of non-ICU patients was more than the normal range. Finally, our patients were divided into two groups: with liver injury and without liver injury. A total of 37 eligible patients were allocated in the non-liver-injury category and 65 participants were assigned in the liver-injury group. All the patients with positive history of liver related diseases, including viral hepatitis, alcoholic hepatitis, liver malignancy or metastasis were excluded. Moreover, after adjustment, none of the factors related to non-alcoholic fatty liver disease (NAFLD) had significant impact on our result. These factors included diabetes mellitus (P=0.39), hypertension (P=0.32), chronic kidney diseases (P=0.65), smoking (P=0.74), alcohol drinking (P=1.00), cardiovascular diseases (P=0.37). Furthermore, for minimizing the effect of NAFLD on the liver injury group, the AST and ALT after viral treatment and discharge time were compared between two group of liver injury and non-liver injury group. No significant difference between them based on the ALT and AST level was reported (P=0.25, P=0.88, respectively). In the current study, a collection of manifestation of COVID-19, co-morbidities, and demographic characteristics were evaluated between these two groups of patients. The mean age in the patients with no liver injury and liver injury group was 54.05±15.90 and 55.74±17.72 years, respectively (P=0.48). The prevalence of male sex in the liver-injury group was higher (64.17%, P=0.21) (table 1). According to statistical analysis, signs, symptoms, and co-morbidities were not significantly different among these two groups (table 1). However, C-Reactive Protein (CRP) Test was significantly higher in the group with liver injury (P=0.01, respectively) (table 1). 

**Table 1:** The statistical analysis results of different characteristics in liver injury

**Characteristics **	**Patients without injury** **(n=37)**	**Patients with injury** **(n=65)**	**P value**
Age (year)	54.05±15.90	55.74±17.72	0.48
Male sex (%)	54.34%	64.17%	0.22
Fever (%)	61.53%	55.76%	0.51
Cough (%)	55.69%	46.15%	0.28
Dyspnea (%)	53.84%	62.74%	0.31
Diarrhea (%)	21.51%	26.92%	0.47
HTN^1^(%)	29.48%	37.25%	0.35
DM^2^ (%)	24.35%	13.72%	0.14
CHD^3^(%)	25.64%	23.52%	0.78
White blood cell count ( 10^9 ^per liter)	5.54±2.91	6.09±2.84	0.30
Lymphocyte count (10^9 ^per liter)	1.15±0.61	1.06±0.58	0.48
Neutrophil count (10^9 ^per liter)	4.37±2.3	4.31±2.19	0.91
admission ALT^4 ^(IU/Liter)	22.08±8	62.42±58.62	0.00
Admission AST^5^ (IU/Liter)	24.51±5.94	64.88±71.79	0.00
ALK-P ^6^(IU/Liter)	183.25±81.24	236.89±199.63	0.69
CRP ^7 ^(mg/Liter)	22.93±18.16	46.26±44.95	0.04
ESR^8 ^(mm/h)	33.45±22.79	36.41±23.48	0.43
Duration of hospitalization(day)	9.65±10.62	7.32±7.08	0.60

^1^ Hypertension ,^2^ Diabetes Mellitus ,^3 ^Coronary heart disease ,^4^ Alanine transaminase ,^5 ^Aspartate transaminase,^6 ^Alkaline Phosphatase,^7^ C-Reactive Protein ,^8^ Erythrocyte Sedimentation rate

Univariate logistic regression revealed the significant associations of AST, ALT, and ALK-P with liver injury in non-ICU COVID-19 patients (P=0.00, OR= 1.18, Cl 95%=1.11-1.25; P=0.00, OR= 1.13, Cl 95%= 1.08-1.18; P=0.04, OR=1.003, Cl 95%= 1.000-1.007). Furthermore, CRP correlated with liver injury in our population, as well (P=0.03, OR=1.009, Cl 95%= 1.000- 1.017) (table 2). Nevertheless, we found no independent predictor for liver injury among non-ICU participants in multivariate analysis. (table 2). The level of CRP significantly correlated with AST (r=0.22, P=0.00). Also, ALT significantly was correlated with neutrophil CRP (r=0.01, P=0.90; r=0.23, P=0.02) (table 3).

**Table 2:** The univariate analysis reported the associated factors and independent predictors for liver injury

**Variables**	**Univariate analysis**		
	**OR**	**95% Cl**	**P**
ALK-P	1.003	1.000-1.007	0.04
CRP	1.009	1.000-1.017	0.03

^ 1^ Alkaline Phosphatase,^2^ C-Reactive Protein

**Table 3:** Spearman’s rank correlation coefficients with the level of AST and ALT in study population

**Characteristics**	**AST**		**ALT**	
	**r**	**Pvalue**	**r**	**Pvalue**
Age	-0.03	0.70	-0.07	0.43
White blood cell count	-0.02	0.87	0	0.99
neutrophil	-0.07	0.51	0.01	0.90
lymphocyte	-0.15	0.14	-0.14	0.17
CRP	0.22	0.00	0.23	0.02
ESR	0.09	0.27	-0.03	0.78
ALK-P	0.04	0.67	0.06	0.53
Hospital duration	0.05	0.58	0.03	0.74

^1^ C-Reactive Protein,^2^ Erythrocyte Sedimentation rate,^3^ Alkaline Phosphatase

## Discussion

The results of the present study showed that no independent variable of laboratory data could independently predict liver injury among patients infected with COVID-19. However, some parameters were associated with liver injury, including C-reactive protein and alkaline phosphatase.

Recently, the correlation of several biomarkers with survival in infected patients with COVID-19 has been considered. These factors include leukopenia, elevated CRP, or ESR, and liver enzymes. It seems that the elevation of these markers is associated with inflammatory pathways that could lead to mortality (6, 11).

Previous studies about severe acute respiratory syndrome coronavirus (SARS-COV) and Middle East Respiratory Syndrome (MERS) reported liver injury due to these infections. These infections significantly affect liver functions and cause necro-inflammation processes (6, 12). The pathophysiology of this damage is unclear. But, different pathways have been introduced for this affection. Some evidence represented that the mechanism of this injury is accelerated via Angiotensin-Converting Enzyme 2 (ACE2) receptors. These receptors cause direct infection of endothelial liver cells with these viruses (13). Other studies reported that SARS-COV could hurt the liver tissue directly via the Caspase-dependent pathway (14). However, some investigations did not find the presence of these agents in liver tissues of deceased patients after autopsy (6). These studies highlight the role of inflammatory pathways and cytokines in liver injury (11). In addition to viral infections, drug toxicity has an important role in liver injury such that some anti-viral agents, including Lopinavir, Ritonavir, and chloroquine cause liver injury (15). Therefore, drug toxicity can affect liver enzyme changes and final results (16). 

Hence, in the present study, the first admission level of the liver enzyme was used for dividing the patients to liver injury and non-liver injury. Additionally, in our study patients were excluded, if they previously received any medications or admitted in other hospital due to COVID-19 or had positive history of chronic liver diseases. Hypertension, high body mass index (BMI), and diabetes mellitus (DM) were numbered as the metabolic syndrome that could predict the risk of non-alcoholic fatty liver diseases (NAFLD) (17). Increasing the fat in the liver and insulin resistance due to diabetes mellitus type 2 (DM2) or obesity could lead to NAFLD. In many cases, this disorder is considered a silent disease, that no elevation of liver enzymes could be detected. However, in nearly most of the patients, this disorder is detected with elevated liver enzymes, in the absence of clinical presentation (18). On the other hand, the most common cause of death in the advanced stage of this disease is because of cardiovascular diseases (19). Nevertheless, in the current study, the results were adjusted as the metabolic syndrome components, and the impact of these predictors of NAFLD that could lead to elevated liver enzymes was omitted. Therefore, it seems, the better perspective of the role of COVID-19 in liver injury and elevation of liver enzymes was provided. Thus, the hepatotoxic effects of COVID-19 medications and fatty liver disease have been eliminated from our study. It is of note that these agents could cause diarrhea, as well (20). In the case of diarrhea, other studies reported the frequency range of 2% to 50% among COVID-19 patients (21). So, it seems that the higher rate of diarrhea in our hospitalization cases was due to anti-viral treatments. 

Surprisingly, COVID-19 pneumonia was accompanied by hepatic involvement in the primary investigation, which focused on COVID-19 characteristics in Wuhan city. The effect of the COVID-19 virus on liver injury is one of the challenging issues since the onset of this disease; because we do not have clear knowledge about the mechanism of liver injury in COVID-19. Several recent descriptive studies have reported that liver enzymes significantly increased due to COVID-19 infection (22). However, most of them just evaluated the liver damage in critical and ill cases and severe form of infection. But, the liver injury characteristics in non-ICU patients have not been investigated carefully. The study by Guan et al. (23) revealed abnormal AST and ALT levels in 22% of patients infected with COVID-19. This number was significantly higher in ICU admitted and ill patients. Cai et al. (24) showed a similar result in their study. It has been evidenced that up to 50% of patients had a degree of liver injury due to Covid-19 infection, and there was a direct correlation between mortality rate and elevated liver enzymes (13). The ALT enzyme could specifically detect more liver injury, but AST could increase in other non-related liver situations (6). 

A new study by Chen et al. showed that of 99 COVID-19 cases with liver injury, only 28% of them show ALT increase. So, we decided to use ALT, AST, and bilirubin to estimate liver injury to increase the sensitivity of our study. In particular, 39.8% of non-ICU patients in our study have an abnormal liver function and entered to liver injury group; this result is similar to those of Zhenyu et al. (25) and XIE et al. (10). In the present research, the number of male patients was more than that of the females. It seems the sexual hormones and X chromosome play a protective role against COVID-19 in the female population; however, the cause is not clear yet (22). Moreover, based on the current study, overall, the patients were at the end of the middle-aged. So, it appears that this infection affects the elderly men population more than other groups. 

Nevertheless, we could find any significant difference in age and gender among liver injury and non-liver injury groups. Although over 20% of the study population had at least one co-morbidity, including HTN, DM2, and CHD, the prevalence of these disorders were not significantly diverse among liver injury and non-liver injury groups. Hence, it seems that despite the negative effects of co-morbidities on the rate of infection and mortality, they do not lead to liver injury (26). 

Despite several studies about the liver injury in ICU-admitted and critically infected patients, there is limited evidence about the related factors with liver injury in non-ICU patients and a mild form of infections. In the present study, it was attempted to find the factors associated with liver injury among laboratory factors, signs and symptoms, demographic characteristics, and underlying disorders in infected patients with COVID-19. Although no independent factor was found among the input variables, it seems that CRP and ALKP were associated with liver injury. however, the degree of computed tomography scan (CTscan) lesions in the lung was the only independent factor which was reported in Xie et al study and there was not any independent association between CRP and liver injury in previous studies(27, 28). The relation of CRP with liver injury supports the inflammatory pathways in liver injury due to COVID-19 infection. 

CRP is an acute-phase reactant synthesized by hepatocytes in response to systemic inflammation. This reactant helps detect or predict outcomes of inflammation. CRP has many pathophysiologic roles in the inflammation process, including recognizing some foreign pathogens, activating the complement system, initiating the elimination of targeted cells, inducing inflammatory cytokines, and stimulating tissue factor in monocyte (11, 29). This marker in acute inflammation increases quickly during 2 days of inflammation. So, it can be considered as an effective predictor in the early stages of the disease. Furthermore, fever and elevated CRP in viral infections are common in this regard. CRP significantly induces chemotaxis and flare activity of respiratory systems (11, 30). Limited similar studies have achieved other items associated with liver injury, including duration of hospitalization or white B cells, count. However, in the present study, no significant differences were found between the two groups according to these items. In this regard, future investigation could help us to achieve a common conclusion for prevention and treatment decisions. 

Our study has several limitations. First, it was a retrospective study and thus we have some missing data in the records of our patients. Second, our sample size was limited and also our investigation was single-center. Moreover, we reported an association between COVID-19 and liver injury without clarifying its cause. Additionally, however, we eliminated a group of patients who have had previous history of chronic liver disease, and also tried to omit the effect of fatty liver on our study by adjusting and modifying fatty liver risk factors such as BMI and other related metabolic factors, but radiologic examination is necessary for exact diagnosis of fatty liver disease. Unfortunately, according to COVID-19 pandemic and medical resource restrictions, we cannot rule out fatty liver by radiologic examination for all of our patients, so this can be another limitation for our study. Furthermore, liver enzymes level after treatment of covid-19 was compared in our study. The report showed that, there was no significant difference between liver injury and non-liver injury after covid-19 treatment. Hence, this data suggests that liver enzyme elevation at the admission time is more likely to occur because of covid-19 than other liver diseases such as fatty liver disease. However, the aforementioned conclusion is not entirely precise. Therefore, more studies, specifically molecular investigation, are needed to determine how the liver injury happened.

In conclusion, liver injury and abnormal liver enzymes are frequent in COVID-19 patients. Liver injury is associated with CRP-level; however, there is no predictor of liver injury in COVID-19 infected patients. Hence, liver injury and liver enzymes must be checked and considered in all COVID-19 patients.
